# Boosting Energy Storage Performance of Glass Ceramics via Modulating Defect Formation During Crystallization

**DOI:** 10.1002/advs.202307011

**Published:** 2023-12-08

**Authors:** Fei Shang, Juwen Wei, Jiwen Xu, Haibo Zhang, Yang Xia, Guisheng Zhu, Kunpeng Jiang, Guohua Chen, Zuoguang Ye, Huarui Xu

**Affiliations:** ^1^ Electronical Information Materials and Devices Engineering Research Center of Ministry of Education Guangxi Key Laboratory of Information Materials and School of Material Science and Engineering Guilin University of Electronic Technology Guilin 541004 China; ^2^ Optics Valley Laboratory Hubei 430074 China; ^3^ Faculty of Chemical Engineering Industrial University of Ho Chi Minh City Ho Chi Minh City 71420 Vietnam; ^4^ School of Materials Science and Engineering State Key Laboratory of Material Processing and Die & Mould Technology Huazhong University of Science and Technology Wuhan 430074 China; ^5^ College of Materials Science and Engineering Zhejiang University of Technology Hangzhou 310014 China; ^6^ Department of Chemistry and 4D LABS Simon Fraser University Burnaby BC V5A 1S6 Canada

**Keywords:** defect modulation, dielectric energy storage, glass ceramics, power density, twin structure

## Abstract

Along with the demand for further miniaturization of high and pulsed power devices, it becomes more and more important to realize ultrahigh recoverable energy storage density (W_rec_) with high energy storage efficiency (η) and ultrahigh discharge energy storage density (W_d_) accompanied by high power density (P_d_) in dielectrics. To date, it remains, however, a big challenge to achieve high W_rec_ or W_d_ in glass ceramics compared to other dielectric energy storage materials. Herein, a strategy of defect formation modulation is applied to form “amorphous‐disordered‐ordered” microstructure in BaTiO_3_‐based glass ceramics so as to achieve a high W_rec_ of 12.04 J cm^−3^ with a high η of 81.1% and an ultrahigh W_d_ of 11.98 J cm^−3^ with a superb P_d_ of 973 MW cm^−3^. This work demonstrates a feasible route to obtain glass ceramics with an outstanding energy storage performance and proves the enormous potential of glass ceramics in high and pulsed power applications.

## Graphical abstract

Defect formation modulated by electric field during crystallization can produce an “amorphous‐disordered (twin)‐ordered” structure resulting in outstanding energy storage performance of glass ceramics with ultra high W_rec_ of 12.04 J cm^‐3^ and W_d_ of 11.98 J cm^‐3^. This strategy displays a possibility of breaking the inverted relationship between dielectric constant and breakdown strength in glass ceramics.

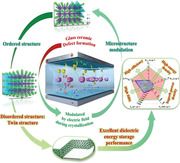

## 1 Introduction

Dielectric capacitors with high power and energy density find important applications in a wide range of power electronics devices.^[^ ^1^ ^]^ It is no doubt that continuously improving energy storage density of dielectrics with high power density is indispensable to further miniaturize high and pulsed power devices, and many strategies were proposed to enhance energy storage capability of dielectric films, such as polymorphic nanodomain,^[^ ^2^ ^]^ superparaelectric relaxor ferroelectrics,^[^ ^3^ ^]^ medium‐entropy relaxors,^[^ ^4^ ^]^ and ion‐bombarded relaxor ferroelectrics,^[^ ^5^ ^]^ etc. However, the inverted relationship between dielectric constant (ε_r_) and dielectric breakdown strength (DBS) in bulk dielectrics becomes an obstacle to continuously enhance energy storage density of dielectrics.^[^ ^6^, ^7^ ^]^ On the other hand, glass ceramics produced by controlled crystallization of amorphous glasses offer the potential of retaining the high dielectric permittivity of ceramics, and the high dielectric breakdown strength and fast charge/discharge rate of glasses, which seems hopeful to break the inverted relationship and thus produce high power and energy density.^[^ ^8^ ^]^ Although many efforts have been put in exploring the methods for enhancing the energy storage density in glass ceramics, such as by introducing nucleating agents like ZrO_2_ or TiO_2_,^[^ ^9^, ^10^ ^]^ glass network modifiers like Na_2_O and K_2_O,^[^ ^11^, ^12^ ^]^ and rare‐earth/transition metal oxide additives like CeO_2_, Sc_2_O_3_, Gd_2_O_3_, La_2_O_3_, Sm_2_O_3_, and Ta_2_O_5_,^[^ ^13^, ^14^, ^15^, ^16^, ^17^ ^]^ the recoverable energy storage density (W_rec_) or measured discharge energy storage density (W_d_) is unfortunately still too low (normally less than 1 J cm^−3^) to meet the requirement of pulsed power technology for dielectrics. One root cause can be attributed to the strong interfacial polarization between glass and crystal grains which will deteriorates the DBS and the energy conversion efficiency (η).^[^ ^18^, ^19^, ^20^ ^]^

It should be noted that the superparaelectric relaxor ferroelectric strategy in dielectric film enlighten the important effect of paraelectric phase on improving dielectric energy storage performance.^[^ ^3^ ^]^ Our previous work demonstrated a new strategy of precipitating paraelectric (cubic) BaTiO_3_ phase from parent glass by traditional thermal treatment to achieve a high DBS in BaTiO_3_ glass ceramic due to similar polarization natures between the paraelectric phase (crystal phase) and the linear dielectric (glass phase).^[^ ^21^ ^]^ Although a high W_rec_ of 3.66 J cm^−3^ with η of ≈70% at 1000 kV cm^−1^ was obtained in this BaTiO_3_ glass ceramic, the potential advantages of glass ceramics, especially in achieving a high DBS, have not been fully explored yet compared to dielectric ceramics.

The crystallization process in glass is a process of phase separation which is controlled either by the classical nucleation and growth mechanism, or by the spinodal decomposition mechanism.^[^ ^22^ ^]^ In either case, diffusion plays an important role and can be influenced by an external electric field applied during crystallization.^[^ ^23^ ^]^ It has been found that the electric field can stimulate nucleation and growth in such glasses as CaO‐Al_2_O_3_‐SiO_2_‐MgO,^[^ ^23^ ^]^ CaO‐B_2_O_3_‐P_2_O_5_,^[^ ^24^ ^]^ and BaO‐TiO_2_‐SiO_2_‐Al_2_O_3_.^[^ ^25^ ^]^ For glass ceramics, a high nucleation and growth rate typically means an enhancement in dielectric constant which in turn may be helpful for improving the dielectric energy storage performance. However, the enhancement in dielectric constant may take place at the cost of deteriorating dielectric breakdown strength due to a stronger interfacial polarization. To date, it is still hard to evaluate the role of electric field assisted crystallization on the energy storage properties of glass ceramics. Thus, it is very necessary to systematically investigate the effects of electric field assisted‐crystallization on the energy storage performance in BaTiO_3_‐based glass ceramics.

In this work, the effects of electric field assisted‐crystallization on the phase structure, microstructure, dielectric properties, interfacial polarization, and energy storage performance are systematically studied in the BaO‐TiO_2_‐Al_2_O_3_‐SiO_2_ glass ceramics crystallized under different electric field strengths of 0, 1, 2, 3, 5, and 7 kV cm^−1^. The corresponding glass ceramic samples are abbreviated as BTAS‐0, BTAS‐1, BTAS‐2, BTAS‐3, BTAS‐5, and BTAS‐7, respectively. Combined with the results of X‐ray photoelectron spectroscopy (XPS), a probable defect formation mechanism with electric field assistance is proposed to explain the reasons for the ultrahigh W_rec_ of 12.04 J cm^−3^ with a high η of 81.1% and the ultrahigh W_d_ of 11.98 J cm^−3^ with a superb P_d_ of 973 MW cm^−3^ at 2000 kV cm^−1^ achieved in BTAS‐3. Moreover, a phenomenological strategy to form “amorphous‐disordered‐ordered” microstructure in glass ceramics is suggested to alleviate the interfacial polarization and boost the energy storage performance, which also shows the possibility of breaking the inverted relationship between ε_r_ and DBS commonly found in glass ceramics.

## 2 Results and Discussion

### 2.1 Phase and Microstructure Analysis of BTAS Glass Ceramics

#### 2.1.1 XRD and Raman Analyses

**Figure 1:**
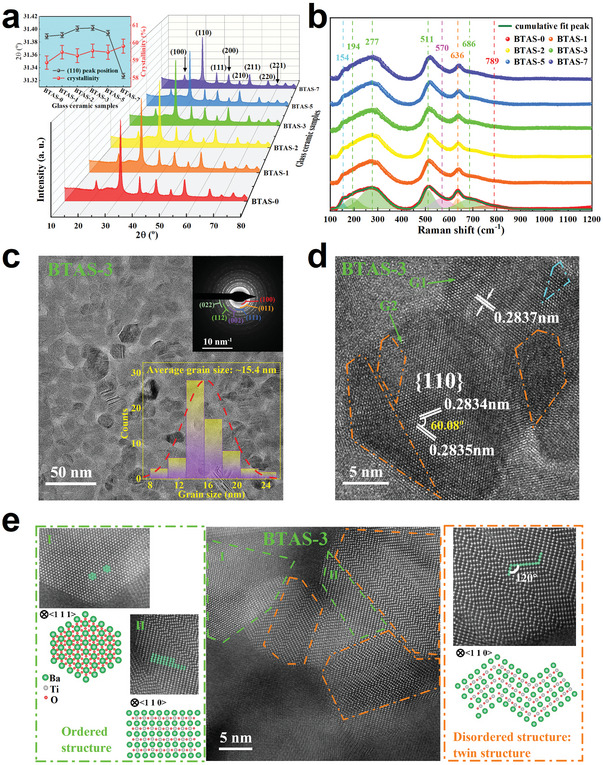
a) XRD patterns of the BTAS glass ceramics crystallized under different electric field strengths. The inset is the variations of the (110) peak position and the crystallinity in different BTAS glass ceramics. b) Raman spectra of the corresponding BTAS glass ceramics at room temperature. c) Morphology of BTAS‐3 glass ceramic observed by HRTEM. The insets are SAED pattern and the grain size distribution histogram. d) Lattice fringes of the BTAS‐3 glass ceramic. e HAADF‐STEM images of the BTAS‐3 glass ceramic together with schematic diagrams of cubic BaTiO_3_ crystal structure under different project vectors.

**Figure** **1**a shows the XRD patterns of the BTAS glass ceramics crystallized under different electric field strengths, where only pure BaTiO_3_ phase with perovskite structure can be observed and the (200) peak ≈45° has no sign of splitting for all the BTAS glass ceramic samples. This means that the electric field strength (≤ 7 kV cm^−1^) applied during crystallization has no obvious impact on the structure of the crystals precipitated from glass matrix. However, from the inset of Figure 1a, the (110) peak position shifts towards to a higher angle slightly, and then back to a lower angle, when the electric field strength is increased from 0 to 7 kV cm^−1^, which indicates that with increasing electric field strength during crystallization the unit cell of the BaTiO_3_ crystals precipitated from glass matrix first shrinks and then expands. Meanwhile, the crystallinity increases only slightly from 58.88 ± 0.38% to 59.81 ± 0.40%, which indicates that the electric field assisted‐crystallization has a limited effect on the crystallinity and thereby on the dielectric constant of the BTAS glass ceramics. To further investigate the influence of the electric field applied during crystallization on the local symmetry, Raman spectra were measured and shown in Figure 1b. As can be seen in Figure 1b, the whole Raman spectra of all the samples are quite similar and can be deconvoluted into several bands located ≈154, 194, 277, 511, 570, 636, 686, and 789 cm^−1^. The details of these deconvoluted bands can be found in our previous work,^[^ ^21^ ^]^ and in this work, we mainly focus on two aspects: one is that all the room temperature Raman spectra lack of the sharp peak ≈305 cm^−1^, indicating that all the BTAS glass ceramics have a cubic BaTiO_3_ phase and the electric field strength (≤ 7 kV cm^−1^) applied does not have significant impact on the phase of crystals, which is consistent with the observation from XRD patterns. Another one is that the deconvoluted band located ≈789 cm^−1^ can be assigned to the symmetric Al‐O^−^ stretching vibration related to the non‐bridging oxygen (NBO).^[^ ^26^, ^27^ ^]^ The intensity of this band is quite low and does not change obviously under different electric field strengths, so it can be inferred that the NBO induced by Al‐O^−^ is not the main cause affecting the energy storage performance of the BTAS glass ceramics.

#### 2.1.2 Microstructure Analysis

It is known that microstructure strongly influences the energy storage performance of glass ceramics. Thus, high resolution transmission electron microscopy (HRTEM) was employed to check the variance of microstructure for the BTAS glass ceramics crystallized under different electric field strengths of 0, 3, and 7 kV cm^−1^, and the results are shown in Figure 1c,d for the BTAS‐3, and Figure S1 (Supporting Information) for the BTAS‐0 and BTAS‐7, glass ceramics. All the samples have similar morphology, with nanocrystal grains embedded in the glass matrix, forming a compact and uniform microstructure. Based on the morphology observed by HRTEM, the grain size distribution histograms of BTAS‐0, BTAS‐3 and BTAS‐7 were calculated and given in the insets of Figure 1c and Figure S1 (Supporting Information). The average grain sizes are ≈15.8, 15.4, and 18.7 nm for BTAS‐0, BTAS‐3, and BTAS‐7, respectively. Due to the small grain size and random orientation, the selected area electron diffraction (SAED) patterns of all the samples display diffraction rings shown in the insets of Figure 1c and Figure S1 (Supporting Information), and these diffraction rings can be indexed to the (100), (110), (111), (200), (211), and (220) crystallographic planes of cubic BaTiO_3_, which provides another evidence for the observed results of XRD patterns and Raman spectra.

Moreover, two interesting phenomena are observed in the lattice fringes of HRTEM, as depicted in Figure 1d and Figure S1 (Supporting Information). One is that the preferred growth direction of nano‐crystals changes from the (110) to (100) crystallographic plane as the electric field strength applied increases from 0 to 7 kV cm^−1^. For BTAS‐0 (seen Figure S1c, Supporting Information), the lattice fringes are parallel due to relatively complete crystal growth plane and the fringe spacings of two nano‐crystal grains are ≈0.2871 and 0.2897 nm, which can be assigned to the (110) crystallographic plane of cubic BaTiO_3_. For BTAS‐3, as can be seen in Figure 1d, the appearance of lattice fringes in some grains (marked by G1) looks like that of BTAS‐0, and the fringe spacing is ≈0.2837 nm. In other grains (marked by G2), however, the lattice fringes look different from that of BTAS‐0, which suggests more than one crystal growth plane. After the fringe spacings of different directions and the angle between them were measured, it is found that the fringe spacings of different directions are quite close (≈0.2834 and 0.2835 nm, respectively) and the angle between them is ≈60°, which indicates that the crystal growth plane in BTAS‐3 still belongs to the {110} crystallographic family. The (110) interplanar spacing of BTAS‐3 is apparently smaller than that of BTAS‐0, which supports the (110) peak position shifting toward to a higher angle, as observed from XRD patterns. For BTAS‐7, shown in Figure S1d (Supporting Information), the lattice fringes spacings of two nano‐crystal grains are ≈0.4028 and 0.4039 nm, which corresponds to (100) crystallographic plane of cubic BaTiO_3_, indicating that the preferential growth crystal plane has changed compared to that of BTAS‐0 and BTAS‐3. Another interesting phenomenon is that Figure 1d and Figure S1 (Supporting Information) also reveal that in all the glass ceramic samples there are some “dark shading” regions which are marked by dash dot polygons with cyan or orange color. These “darker” domains are generally considered to be induced by local defects, such as oxygen vacancies.^[^ ^28^ ^]^ It should be noted that in BTAS‐3 the most “dark shading” regions marked by orange dash dot polygons look quite different from those marked by cyan dash dot polygons in BTAS‐0 and BTAS‐7. The lattice fringes in the former regions seem fuzzy and disordered, whereas the latter regions contain continuous lattice fringes indicating that these regions are structurally coherent with the other “brighter” area in nano‐crystal grains. In order to further clarify the domain structure in nano‐crystals of the BTAS‐3 glass ceramic, atomic‐scale high‐angle annular darkfield (HAADF) images were collected and shown in Figure 1e based on atom‐resolved STEM. In Figure 1e, two domain structures are observed, marked by green and orange dash dot polygons respectively. The regions labelled by I and II show two different appearances, and the enlarged views combined with schematic diagram of cubic BaTiO_3_ crystal structure under <111> and <110> project vectors are shown on the left side of Figure 1e. The green circle dots represent A‐site cation (Ba^2+^ ion). It can be seen that region I and II represent an ordered structure in nano‐crystals. Whereas, from the right side of Figure 1e, the enlarged view of the region marked by orange dash dot polygons shows a “zig‐zag” twin structure in one nano‐crystal and the included angle of the zig‐zag shape is ≈120°. Compared to the schematic diagram of cubic BaTiO_3_ crystal with twin structure under <110> project vector, it can be concluded that the twin structure is originated from the preferential growth crystal planes from the {110} crystallographic family, such as (110) and (101), which is also evidenced as the observed results from lattice fringes of HRTEM (Figure 1d). Thus, the observation of HAADF‐STEM indicates that the “amorphous‐disordered‐ordered” microstructure does exist in the BTAS‐3 glass ceramic and the disordered structure is a twin structure. Based on the above discussion, it is reasonable to deduce that the main effect of the electric field applied during crystallization is to modulate defects formation so as to influence the microstructure of glass ceramics and thereby the energy storage performance.

### 2.2 Possible Defect Formation Mechanism

**Figure 2:**
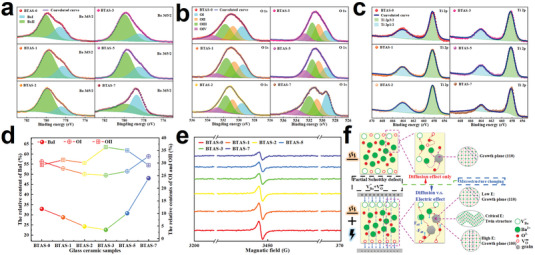
a) XPS high resolution spectra deconvoluted results of the Ba 3d5/2 peaks. b) XPS high resolution spectra deconvoluted results of the O 1s peaks. c) XPS high resolution spectra deconvoluted results of Ti 2p peaks. d) The relative contents of BaII, OI and OII for different BTAS glass ceramic samples based on the deconvoluted results of Ba 3d5/2 and O 1s peaks. e) The first‐derivative EPR spectra of BTAS‐0, 1, 2, 3, 5, and 7 glass ceramics. f Sketches of defect formation processes without and with electric field assistance during crystallization.

**Figure** **2**a–c presents the high resolution X‐ray photoelectron spectroscopy (XPS) results of the BTAS glass ceramics crystallized under different electric field strengths. As can been seen in both Figure 2a,b, with the electric field strength increasing, the profiles of the Ba 3d5/2 and O 1s peaks change apparently, which indicates that the electric field does have some impact on the bonding and chemical environments during crystallization and thereby on the defect formation in the glass ceramics. The Ba 3d5/2 state can be deconvoluted into two peaks, one at ≈778.66 eV marked as BaI which is assigned to Ba^2+^ ion in the perovskite structure of BaTiO_3_, and the other one at ≈779.99 eV marked as BaII which is related to the Ba‐site in the glass matrix.^[^ ^29^, ^30^, ^31^, ^32^ ^]^ According to the deconvoluted results of the Ba 3d5/2 state, as depicted in Figure 2a, the relative amount of BaI (BaI / (BaI+BaII)) decreases firstly and then increases when the electric field strength increases from 0 to 7 kV cm^−1^. The variation of the BaI relative amount for each BTAS glass ceramic sample is given in Figure 2d. The O 1s peaks can be deconvoluted into four peaks, marked by OI, OII, OIII, and OIV, respectively. The OI peaks ≈529.49 eV can be assigned to the lattice oxygen in the BaTiO_3_ crystal grains,^[^ ^30^, ^33^ ^]^ the OII peaks at ≈530.70 eV can be attributed to NBO in glass matrix,^[^ ^29^ ^]^ the OIII peaks around 531.68 eV can be assigned to the bridging oxygen (BO) in glass matrix^[^ ^29^ ^]^ and the OIV peaks located at larger than 532.00 eV are related to the chemical absorbed ‐OH due to ethanol cleaning after grinding and polishing, which are excluded from the estimation of the relative amounts of OI and OII.^[^ ^30^, ^33^ ^]^ It should be noted that due to a quite low Raman scattering intensity of Al‐O^−^ stretching vibration (Figure 1b), the OII peaks should mainly consist of NBO associated with Ba = O or Ba‐O‐Q^[i]^ (Q^[i]^ represents differently coordinated units in the glass matrix). Based on Figure 2b, the relative contents of OI (OI / (OI+OII+OIII)) and OII (OII / (OI+OII+OIII)) for the different BTAS glass ceramics were calculated and plotted in Figure 2d. The relative amount of OI has a similar changing trend to that of BaI, i.e., the contents of Ba and O in the BaTiO_3_ nanocrystals synchronously increase or decrease, whereas the relative amount of OII has an opposite variation trend to those of BaI and OI. Generally, it is widely believed that in BaTiO_3_‐based glass ceramics the valence state of Ti ion can easily change from Ti^4+^ to Ti^3+^, which could degrade DBS and thereby the energy storage performance. However, in this work, no sign of Ti^3+^ can be found, but only Ti^4+^ exists in the BTAS glass ceramics, even in BTAS‐0, because the high resolution Ti 2p spectra of all the BTAS glass ceramics can be fitted well with a single pair of peaks: the Ti 2p3/2 peaks of BTAS‐0, BTAS‐1, BTAS‐2, BTAS‐3, BTAS‐5 and BTAS‐7 centered at 458.34 eV with an FWHM of 1.53 eV, 458.35 eV with an FWHM of 1.62 eV, 458.32 eV with an FWHM of 1.54 eV, 458.31 eV with an FWHM of 1.61 eV, 458.24 eV with an FWHM of 1.57 eV and 458.18 eV with an FWHM of 1.36 eV, respectively and Ti 2p1/2 peaks determined by adding spin‐orbit splitting (5.72 eV) to the corresponding Ti 2p3/2 peaks, as shown in Figure 2c. The Ti 2p3/2 peak located around 458.30 eV is characteristic of the Ti^4+^ state in the BaTiO_3_ ceramics.^[^ ^34^ ^]^ Moreover, the Ti 2p3/2 peaks of BTAS‐5 and BTAS‐7 shift to a lower binding energy apparently, which is related to an increasing Ti‐O distance.^[^ ^35^ ^]^ With the Ti‐O distance increasing, the interplanar spacing of the (110) plane also increases, which is consistent with the observation from the XRD patterns. Besides, all the BTAS glass ceramics possess high optical transmittance in the visible light region and show yellowish color, for example, the average transmittance of the BTAS‐3 glass ceramic in the 450–700 nm wavelength range is ≈70% (shown in Figure S2, Supporting Information, and the inset is the photo of the BTAS‐3 glass ceramic), which gives another evidence for only Ti^4+^ existing in the BTAS glass ceramics.

For binary metal oxides, such as BaO and TiO_2_, the Schottky defect formation energy of $V_{Ba}^{\prime \prime }+V_{O}^{\cdot \cdot }$ is lower than that of ${V^{\prime \prime \prime \prime }}_{Ti}+2V_{o}^{\cdot \cdot }$.^[^ ^36^, ^37^, ^38^ ^]^ Besides, the calculated activation energies for the diffusion of Ti, Ba and O are 15.26, 3.45, and 0.76 eV, respectively.^[^ ^36^, ^37^ ^]^ Meanwhile, considering that only Ti^4+^ state exists in the BTAS glass ceramics, it is reasonable to believe that for the BTAS parent glass a partial Schottky defect reaction occurs during crystallization to produce the $V_{Ba}^{\prime \prime }+V_{O}^{\cdot \cdot }$ defects first on the surface of the glass, while Ti is assumed to be fixed in the glass matrix. In order to verify the above assumptions, electron paramagnetic resonance (EPR) was employed to check the possible defects in the BTAS glass ceramics, shown in Figure 2e. For each BTAS glass ceramic, there is only one singlet signal observed at *g* = 2.003 observed by EPR, which is attributed to an unpaired electron trapped by a pair of barium and oxygen vacancies,^[^ ^39^, ^40^, ^41^, ^42^ ^]^ i. e., the $V_{Ba}^{\prime \prime }+V_{O}^{\cdot \cdot }$ defects do exist in the BTAS glass ceramics. Moreover, the EPR spectrum of parent glass has also been checked, shown in Figure S4 (Supporting Information). And there is no any EPR signal observed in the whole spectrum, indicating that the $V_{Ba}^{\prime \prime }+V_{O}^{\cdot \cdot }$ defects should be formed during crystallization. Thus, the defects formation process during crystallization with and without electric field assistance can be pictured as follows: (1) Without electric field assistance during the crystallization of BTAS parent glass, parts of Ba and O ions will participate in nucleation and growth, while the other Ba and O ions in glass matrix diffuse to the surface of the parent glass due to the partial Schottky defect reaction, as depicted in Figure 2f. This leads to much lower contents of Ba^2+^ (BaII) and O^2−^ (OII) in glass matrix, and thereby the relative contents of BaI and OI are relatively high, as observed in XPS (seen in Figure 2d). (2) With electric field assistance during the crystallization of BTAS parent glass, the effective electric field (E_eff_) in glass generates an electric field force on the Ba^2+^ (F_eff_) and O^2−^ (‐F_eff_) ions, which inhibits Ba^2+^ and O^2−^ diffusion to the surface to form a competing relationship with the diffusion effect, as displayed in Figure 2f. When the strength of electric field is low (such as 1 and 2 kV cm^−1^ in this work), the diffusion effect still plays a dominant role during crystallization, but due to the restrictive effect of E_eff_, more Ba^2+^ and O^2−^ are left in the glass matrix and thus the relative contents of BaI and OI decrease. When a “critical” electric field (i.e., 3 kV cm^−1^ in this work) is applied during crystallization, E_eff_ is strong enough to balance the diffusion effect, and many Ba^2+^ and O^2−^ ions are restricted in the glass matrix. Thus, the relative contents of BaI and OI continuously decrease, as observed by XPS. In this situation, parts of these Ba^2+^ and O^2−^ ions restricted in the glass matrix by electric field may participate in the growth of crystal grains precipitated from the glass matrix, but due to the uncomplete elimination of diffusion effect some “disordered” structure appears in the crystal growth (as seen in Figure 1e), as evidenced by the larger FWHM of the BaI peak in the BTAS‐3 glass ceramic based on the deconvoluted results of XPS shown in Figure 2a. When the electric field strength is higher than the “critical” electric field (such as 5 and 7 kV cm^−1^ in this work), E_eff_ is strong enough to overcome the diffusion effect and Ba^2+^ and O^2−^ will be “fixed” in the glass matrix. In this case, these fixed Ba^2+^ and the O^2−^ ions participate in the nucleation and growth of crystal grains, and thus the relative contents of BaI and OI increase apparently and those of the BaII and OII decrease. Besides, sufficient Ba and O in the glass matrix are also helpful for “perfect” growth of crystal grains resulting in the slightly increasing crystallinity observed by XRD (Figure 1a) and the change in the preferred growth orientation confirmed by the lattice fringes of HRTEM in BTAS‐7 (Figure S1d, Supporting Information). Overall, from the perspective of defect formation process, the above‐discussed mechanism can qualitatively explain the changing trends of the relative contents of BaI, OI, and OII, as observed from the XPS spectra (shown in Figure 2d). Moreover, energy‐dispersive X‐ray spectroscopy (EDXS) element mapping images shown in Figure S3 (Supporting Information) demonstrate that in BTAS‐7 the Ba element is distributed mainly in the grains with clear boundaries in the same way as the Ti element, whereas in BTAS‐3 the Ba element is apparently dispersed with blurring boundaries, which gives another supporting evidence for the defect formation process discussed above.

### 2.3 Dielectric Properties, Interfacial Polarization, and Electrical Relaxation Process of BTAS Glass Ceramics

#### 2.3.1 Dielectric Properties

**Figure 3:**
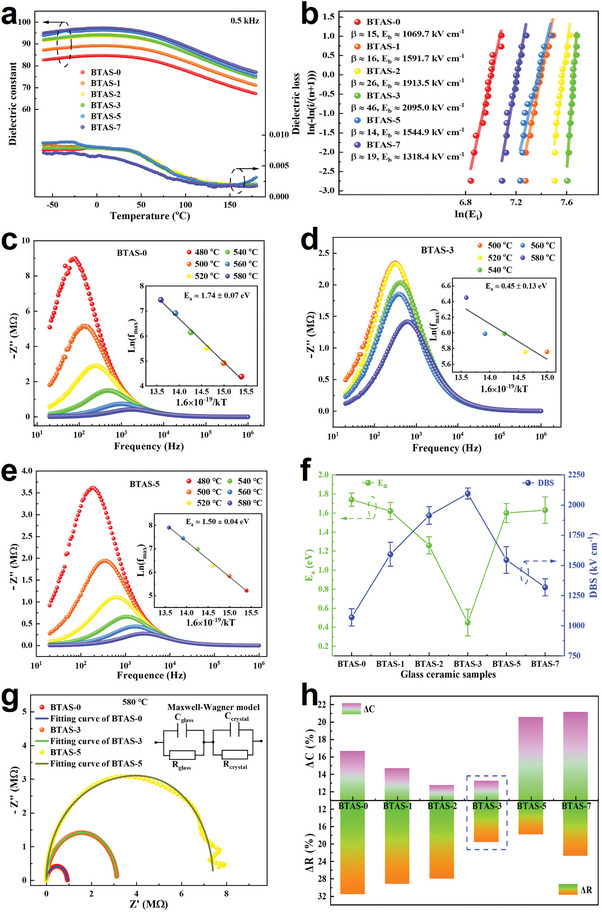
a) Temperature dependent dielectric constant and loss measured at the frequency of 0.5 kHz. b) Weibull distribution of dielectric breakdown strength E_b_ of the glass ceramics with a thickness of 0.05 ± 0.01 mm at room temperature. ‐Z′′ versus frequency curves at different temperatures of c) BTAS‐0, d) BTAS‐3, and e) BTAS‐5. f) E_a_ and DBS as a function of various electric field strengths applied during crystallization. g) Cole‐Cole plots of BTAS‐0, BTAS‐3, and BTAS‐5 at 580 °C. The inset is the equivalent circuit of “brickwork” model. h Variations of ΔR and ΔC for the BTAS glass ceramics.

**Figure** **3**a gives the temperature dependent dielectric constant and loss for BTAS‐0, BTAS‐1, BTAS‐2, BTAS‐3, BTAS‐5, and BTAS‐7. All the dielectric constant‐temperature (ε_r_ – T) curves display a visible paraelectric behavior,^[^ ^43^ ^]^ with a quite low dielectric loss ranging from ≈0.002 to 0.008 in the temperature span of −60 to 180 °C. With the electric field strength applied during crystallization increasing, the dielectric constant increases slightly, for example, the ε_r_ of BTAS‐7 (≈96 at 25 °C) is increased only by ≈8% compared to that of BTAS‐0 (ε_r_ ≈88), which can be ascribed to the slight increase in crystallinity. The E_b_ values are shown in Figure 3b based on the Weibull distribution, and all the β parameters of the different BTAS glass ceramics are larger than 14, which demonstrates a good data consistency. The E_b_ values increase substantially when the electric field was applied during crystallization, which is very helpful for improving the energy storage performance. It should be noted that along with the significant increasing of E_b_ the ε_r_ does not decrease but slightly increase, apparently breaking the inverted relationship between ε_r_ and DBS in glass ceramics. An ultrahigh value E_b_ = 2095.0 kV cm^−1^ is obtained in BTAS‐3 with ε_r_ of ≈91. More recently, it has also been found that the twin boundary is helpful to enhance the E_b_ value of BaTiO_3_‐(Bi_0.5_Na_0.5_)TiO_3_‐CaZrO_3_ ternary solid solution ceramics.^[^ ^44^ ^]^ Thus, this implies that in our work the defect formation modulated by electric field applied during crystallization so as to form twin (disordered) structure has an obviously favorable impact on the dielectric breakdown strength of glass ceramics. In order to better understand the underlying mechanisms of this impact, impedance spectroscopy was utilized to analyze the electrical microstructure of the BTAS glass ceramics.

#### 2.3.2 Interfacial Polarization and Electrical Relaxation Process

For glass ceramics, the interfacial polarization between glass matrix and precipitated nano‐crystals is originated from dielectric mismatch leading to space charge accumulation at the interface of two different types of dielectrics when an external electric field is applied. Generally, the relaxation of space charge determines the magnitude of interfacial polarization, that is, a high relaxation rate (short relaxation time) will alleviate the interfacial polarization, which may be useful for the DBS improvement in glass ceramics. It is known that relaxation time is related to the activation energy (E_a_) during relaxation process, and E_a_ can be estimated by analyzing the relaxation frequencies at different temperatures using the Arrhenius relationship: *f*  = *f*_0_ *exp*( − *Ea*/*kT*), where f is the frequency at which the imaginary component (Z′′ or M′′) reaches peak value in the plot of Z′′ or M′′ versus frequency, f_0_ is the frequency factor, k is the Boltzmann constant and T is the temperature at which the complex impedance testing is carried on.^[^ ^45^ ^]^ For the insulators with a large resistance, the complex impedance (Z* = Zʹ‐jZ′′) should be used to analyze the electrical responses.^[^ ^46^ ^]^ Thus, for the BTAS glass ceramic samples crystallized under different electric field strengths, the plots of ‐Z′′ versus frequency at different temperatures were used to evaluate the activation energy values, as shown in Figure 3c–e and Figure S5a–c (Supporting Information), where the insets give the plots of Ln(f_max_) versus 1.6×10^−19^/kT with linear fitting. It can be seen that as the strength of electric field increases the E_a_ decreases first and then increases, while the DBS value shows a contrary changing trend, as depicted in Figure 3f. When the strength of electric field increases from 0 to 7 kV cm^−1^, the E_a_ value firstly decreases from 1.74 ± 0.13 eV in BTAS‐0 to 0.45 ± 0.13 eV in BTAS‐3, and then increases to 1.63 ± 0.14 eV in BTAS‐7, whereas the corresponding DBS value increases apparently from 1069.7 ± 71.1 kV cm^−1^ to 2095.0 ± 45.8 kV cm^−1^ and then deceases to 1318.4 ± 70.4 kV cm^−1^. The value of E_a_ characterizes the interfacial mobility in the defects, and a lower E_a_ represents a more favorable space charge spreading process which is beneficial for mitigating the interfacial polarization, thereby greatly improving the DBS value of glass ceramics.^[^ ^18^, ^47^ ^]^

Furthermore, to identify the electrical response in the BTAS glass ceramics, the Cole‐Cole plots (‐Z′′ versus Zʹ) of the samples at 580 °C are shown in Figure 3g and Figure S5d (Supporting Information). At first glance, except for BTAS‐7, all the other BTAS glass ceramics show a nearly perfect semi‐circle in the Cole‐Cole plot, which indicates one bulk relaxation process in the BTAS glass ceramics. This seems to contradict with the fact that there are two phases in a glass ceramic, i.e., glass and crystal phases. To understand this phenomenon, it should be kept in mind that the precipitated crystal phase is cubic BaTiO_3_ in the BTAS glass ceramics. It is known that cubic BaTiO_3_ is a paraelectric phase, and its polarization process is similar to that of a linear dielectric (glass phase). This eliminates the obvious difference in the polarization relaxation processes between the glass and crystal phases and can reduce the interfacial polarization. Moreover, the arcs in the Cole‐Cole plots of BTAS‐0, 1, 2, 3, and 5 are actually not perfect, but slightly depressed semi‐circles, the center of which is below the Zʹ axis, indicating that there is still some dielectric mismatch between the two phases in the BTAS glass ceramics. Based on the microstructure observed by TEM, a “brickwork” model which is commonly used in electro‐ceramics,^[^ ^48^ ^]^ was utilized to fit the Cole‐Cole plot to further clarify the dielectric mismatch in the BTAS glass ceramics. The equivalent circuit of “brickwork” model consists of two parallel RC circuits in series, as shown in the inset of Figure 3g. Each single parallel RC element represents the contribution from the glass or crystal phase, so the time constant of the glass and crystal phase can be written as τ_*glass*_ = *R_glass_* *C_glass_* and τ_*crystal*_ = *R_crystal_* *C_crystal_*, where τ is the time constant, R is the resistance, C is the capacitance and the footnote represents different phases. If τ_glass_ is equal to τ_crystal_, the dielectric mismatch between the two phases disappears, that is, the electrical transport characteristics tend to be consistent throughout the glass ceramics, resulting in the elimination of interfacial polarization. Apparently, a larger difference of resistance or/and capacitance between the glass and crystal phases corresponds to a larger variation of time constant. Thus, for the convenience of the following discussion, we define the discrepancy rates of resistance and capacitance between the glass and crystal phases as: Δ*R*  = |*R_crystal_* − *R_glass_* |/(*R_glass_* + *R_crystal_*) and Δ*C*  = |*C_crystal_* − *C_glass_* |/(*C_glass_* + *C_crystal_*) , to describe the degree of mismatch between the glass and crystal phases. The values of R_glass_, R_crystal_, C_glass_ and C_crystal_ can be extracted from fitting the Cole‐Cole plot by using Zview software based on the equivalent circuit of “brickwork” model and the fitting curves for the BTAS glass ceramic samples are displayed in Figure 3g and Figure S5d (Supporting Information). Then, the ΔR and ΔC values of the different glass ceramic samples are calculated and shown in Figure 3h to illustrate the influence of the electric field strength on the dielectric mismatch. From Figure 3h, both ΔR and ΔC first decrease and then increase with increasing electric field strength. First, it is known that if the difference in the two time constants is originated from the difference in capacitance, then the arcs are well resolved in the impedance spectrum and if these time constants differ as a result of the difference in resistance, the arcs can be resolved in the modulus spectrum.^[^ ^49^ ^]^ As depicted in Figure 3h, for the BTAS‐5 and 7 glass ceramics, the dielectric mismatch is mainly due to the ΔC value with reference to the ΔR value, so in the impedance spectrum the deformation of semi‐circle is observed, especially for the BTAS‐7 glass ceramics (as shown in Figure 3g; Figure S5d, Supporting Information), whereas for the BTAS‐0, 1, 2, and 3 glass ceramics, the main cause of dielectric mismatch can be attributed to the ΔR value, giving rise to the trailing arc appearing in the modulus spectrum of the BTAS‐0 glass ceramics (see Figure S6a, Supporting Information). Thus, it is a viable method to evaluate the dielectric mismatch in the BTAS glass ceramics by using the ΔR and ΔC values in the BTAS glass ceramics. Secondly, the lowest ΔR and ΔC values were obtained in the BTAS‐3 glass ceramics (marked by blue dashed box in Figure 3h), which means that the dielectric mismatch between the glass and crystal phases in the BTAS‐3 glass ceramics is the least. In this case, the peak frequencies of the ‐Z′′ and M′′ versus frequency plots should be close.^[^ ^50^, ^51^ ^]^ The plots of ‐Z′′ and M′′ versus frequency of the BTAS glass ceramic samples are given in Figure S6b–g (Supporting Information). Although in the all BTAS glass ceramic samples the differences in the peak frequencies between the ‐Z′′ and M′′ versus frequency plots does not show an apparent trend, the mismatch of the peak frequency found in the BTAS‐3 glass ceramic is still the smallest, which indicates that the BTAS‐3 glass ceramic has the minimum dielectric mismatch. Thus, the lowest dielectric mismatch combined with the lowest E_a_ value is responsible for the highest DBS value of 2095.0 ± 45.8 kV cm^−1^ in the BTAS‐3 glass ceramics. Thirdly, from the XPS analysis and TEM results (see Figures 1 and 2), it is reasonable to state that the changing trend of dielectric mismatch including the E_a_ values may be attributed to the changes of relative Ba^2+^ distribution in the glass and crystal grains caused by partial Schottky defect formation modulated by the electric field applied during crystallization. Furthermore, the “disordered” structures observed in BTAS‐3 provides a valuable hint that these “disordered” structures existing between the glass (amorphous structure) and crystal (ordered structure) phases may be the most important factor acting as a “buffer layer” to improve interfacial polarization and thus to enhance energy storage performance.

### 2.4 Dielectric Energy Storage Performance of BTAS Glass Ceramics

#### 2.4.1 The Recoverable Energy Storage Density (W_rec_) and Energy Storage Efficiency (η)

**Figure 4:**
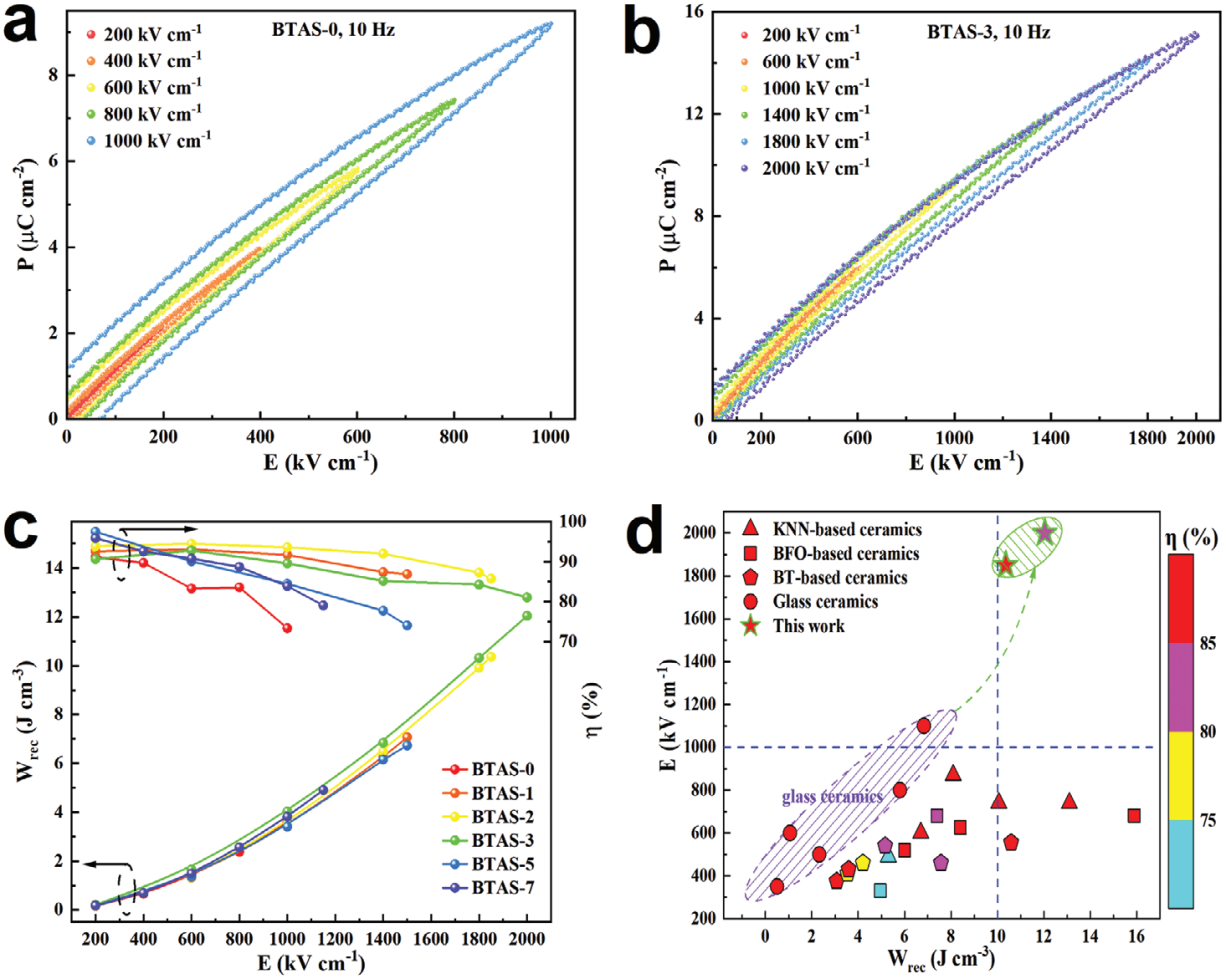
P‐E loops at a frequency of 10 Hz of a) BTAS‐0, b) BTAS‐3 glass ceramics with a thickness of 0.05 ± 0.01 mm and an electrode diameter of 1.5‐2.0 mm. c) W_rec_ and η of the different BTAS glass ceramic samples. d) A comparison of energy storage performance of the BTAS‐2 and BTAS‐3 glass ceramics with the other glass ceramics and dielectric ceramics so far reported. KNN, BFO and BT represent K_0.5_Na_0.5_NO_3_, BiFeO_3_ and BaTiO_3_, respectively.

To evaluate the performance of energy storage properties, P‐E loops were displayed for the BTAS glass ceramics crystallized under different electric field strengths, as depicted in **Figure** **4**a,b and Figure S7a–d (Supporting Information). Based on the results of P‐E loops, the W_rec_ values with η of BTAS‐0, BTAS‐1, BTAS‐2, BTAS‐3, BTAS‐5 and BTAS‐7 are found to be 3.60 J cm^−3^ with 73.4% at 1000 kV cm^−1^, 7.07 J cm^−3^ with 86.9% at 1500 kV cm^−1^, 10.36 J cm^−3^ with 85.8% at 1850 kV cm^−1^, 12.04 J cm^−3^ with 81.1% at 2000 kV cm^−1^, 6.73 J cm^−3^ with 74.1% at 1500 kV cm^−1^, and 4.91 J cm^−3^ with 79.1% at 1150 kV cm^−1^, respectively (displayed in Figure 4c). Apparently, all the samples crystallized with electric field assistance exhibit a better energy storage performance than that crystallized without electric field assistance, which is mainly attributed to the enhancement of DBS at the same time of keeping high ε_r_. This indicates that electric field assisted crystallization is an effective method to enhance the energy storage density and efficiency simultaneously by modulating defect formation to alleviate interfacial polarization in the BTAS glass ceramics. The optimum electric field strengths applied during crystallization, namely 2 and 3 kV cm^−1^, can achieve much better energy storage densities with high efficiencies of 10.36 J cm^−3^ with 85.8% and 12.04 J cm^−3^ with 81.1%, respectively, which represents a very strong energy storage performance compared to many dielectric ceramics so far reported, such as KNN‐based ceramics,^[^ ^52^, ^53^, ^54^, ^55^, ^56^ ^]^ BFO‐based ceramics,^[^ ^57^, ^58^, ^59^, ^60^, ^61^ ^]^ BT‐based ceramics,^[^ ^62^, ^63^, ^64^, ^65^, ^66^, ^67^ ^]^ and other glass ceramics,^[^ ^68^, ^69^, ^70^, ^71^, ^72^ ^]^ as shown in Figure 4d.

#### 2.4.2 Charge‐Discharge Energy Storage Performance

**Figure 5:**
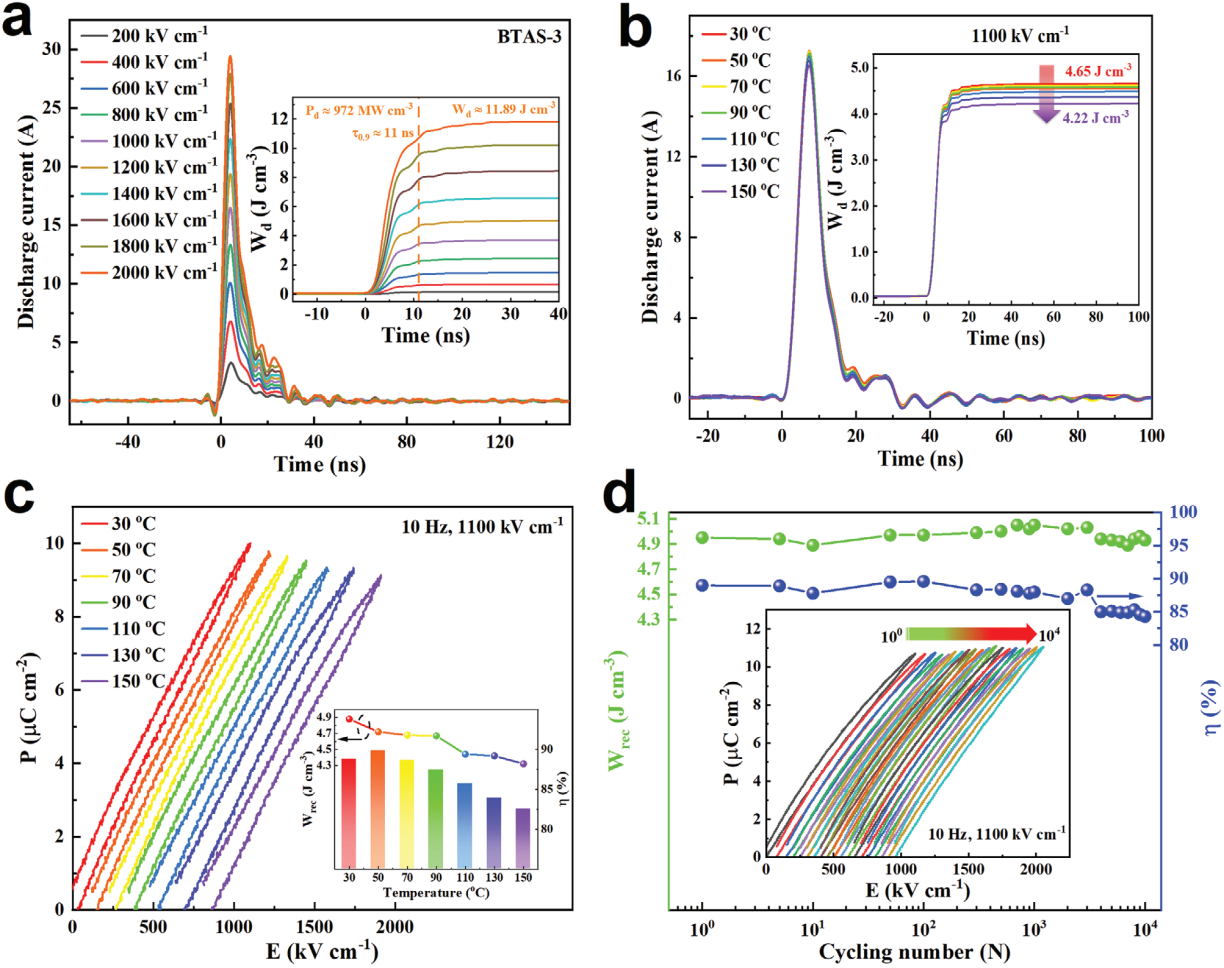
a) The time dependent curves of discharge current of the BTAS‐3 glass ceramic with a thickness of 0.05 ± 0.01 mm and an electrode diameter of 1.5‐2.0 mm. The inset corresponds to the W_d_ versus time curve and P_d_ value. b) Discharge behavior of the BTAS‐3 glass ceramic in the temperature range of 30–150 °C. The inset displays the corresponding W_d_ value. c) P‐E loops of the BTAS‐3 glass ceramic at different temperatures in the range from 30 to 150 °C. The inset gives the temperature dependence of W_rec_ and η. d) The W_rec_ and η values of the BTAS‐3 glass ceramic as a function of the cycling number at room temperature. The inset shows the typical P‐E loops after various cycling numbers.

For high and pulsed power applications, high voltage direct current (HVDC) charge‐discharge behavior characterized by discharge energy density (W_d_) and power density (P_d_) is also a very important parameter to evaluate dielectric energy storage performance. A resistor‐inductance‐capacitor (RLC) charge‐discharge platform with a load resistance of 200 Ω was utilized to record the discharge current as a function of time by using a current meter and an oscilloscope. The W_d_ and P_d_ can be calculated by $W_{d}=\int i{\left(t\right)}^{2}Rdt/V\;$ and $P_{d}=W_{d}^{0.9}/\tau_{0.9}\;$,^[^ ^73^ ^]^ where i(t) is the measured discharge current as a function of time, R is the load resistance, V is the sample volume, $W_{d}^{0.9}$ is the discharged energy in the load resistance to reach 90% of the final value during discharge process and τ_0.9_ is defined as the time required to release 90% of the stored energy. The curves of discharge current versus time of the BTAS glass ceramics are presented in **Figure** **5**a and Figure S8 (Supporting Information), and the corresponding W_d_ and P_d_ values are shown in the insets. For all the samples, only one pulsed current peak is observed under each charging electric field strength, displaying the typical charge‐discharge feature of dielectrics. From the insets of Figure 5a and Figure S6 (Supporting Information), it can be found that the BTAS glass ceramics crystallized with electric field assistance not only possess high W_rec_ and η (shown in Figure 4c), but also exhibit an impressive charge‐discharge energy storage performance. Especially, ultrahigh W_d_ with superb P_d_ values of 11.89 J cm^−3^ with 973 MW cm^−3^ are obtained for the BTAS‐3 glass ceramic, indicating that it has a great potential for high and pulsed power applications. Furthermore, the charge‐discharge energy storage performance of BTAS‐3 glass ceramics with large electrode diameter has also been checked to assess the possibility of mass production, shown in Figure S9 (Supporting Information). From the Figure S9 (Supporting Information), even if the electrode diameter is enlarged to 5 mm, the max DC charging electric field strength can also reach up to 2000 kV cm^−1^ and the corresponding W_d_ is ≈12.11 J cm^−3^. When the electrode diameter is enlarged to 7 mm, the max DC charging electric field strength is 1400 kV cm^−1^ and the corresponding W_d_ is about 6.66 J cm^−3^. It indicates that BTAS‐3 glass ceramic has promising potential of mass production in real applications.

#### 2.4.3 Temperature Stability and Fatigue Behavior

From the perspective of practical applications, temperature stability and fatigue behavior are also studied in the BTAS‐3 glass ceramic, and the results are shown in Figure 5b–d. From Figure 5b,c, it can be seen that the BTAS‐3 glass ceramic demonstrates a good temperature stability in the temperature range of 30–150 °C under a high charge electric field strength of 1100 kV cm^−1^, with a minor variation of W_d_ from 4.65 to 4.22 J cm^−3^ in the temperature range of 30–150 °C and with small variations of 11.5% and 7.1% for W_rec_ and η respectively. Besides, the BTAS‐3 glass ceramic also exhibits a good fatigue behavior at a high charge electric field strength of 1100 kV cm^−1^ at room temperature. From Figure 5d and the inset, it can be seen that after 10^4^ cycles the W_rec_ does not show any decrease and η only decreases slightly from 89.0% to 84.3%. Thus, the outstanding temperature stability and fatigue behavior indicate that the BTAS‐3 glass ceramic is most suitable for practical applications.

## 3 Conclusions

The electric field assisted crystallization provides an effective method to substantially boost dielectric energy storage performance of the BTAS glass ceramics. The electric field applied during the crystallization in glass ceramics weakens the diffusion of Ba^2+^ and O^2−^ ions due to partial Schottky defect formation on the surface of glass ceramics, then affects the relative distribution of Ba^2+^ ion between the glass phase and crystal grains, and thereby influences the growth orientation of grains. An appropriate electric field strength applied during crystallization can introduce an “amorphous‐disordered‐ordered” microstructure in the glass ceramics, which in turn can greatly relieve the interfacial polarization and thus significantly enhance the DBS while keeping a high dielectric constant (ε_r_ ≈91). Consequently, a high W_rec_ of 12.04 J cm^−3^ with a high efficiency of 81.1% and an ultrahigh W_d_ of 11.98 J cm^−3^ with a superb P_d_ of 973 MW cm^−3^ are achieved together with a good temperature stability and a strong fatigue resistance in the BTAS glass ceramics. This outstanding performance indicates that the BTAS glass ceramics are promising candidate for high pulsed power and dielectric energy storage applications.

## 4 Experimental Section

### Glass Ceramics Fabrication

The glass ceramics with the parent glass composition (mol%) of 42BaO‐30TiO_2_‐6Al_2_O_3_‐22SiO_2_ was fabricated by a melt‐quenching‐crystallization technique. The detailed experimental conditions of parent glass preparation can be found in the previous work.^[^ ^21^ ^]^ The prepared parent glass with a thickness of 2 mm was then cut into pieces with square shape of 1 cm × 1 cm and subsequently crystallized by an electric field assisted thermal treatment at the nucleation temperature of 690 °C for 2 h and the crystallization temperature of 875 °C for 2 h. The nucleation and crystallization temperature were decided by the DSC curve of parent glass shown in Figure S10 (Supporting Information) (STA449C, Netzsch, Germany). The electric field assisted thermal treatment equipment was home‐made. Two parallel platinum sheets with a diameter of 8 cm were used as electrodes with the spacing of 2.5 mm. The electric field strength between the two electrodes was controlled by a HVDC source (DW‐P303‐1ACDFO, Dongwen high voltage power supply (Tianjin) Co., Ltd, China).

### Characterization of Phase, Microstructure, Valence State, and EPR

The phase structures were determined by X‐ray diffraction (2theta range: 10–80^o^, step: 0.01^o^, Smartlab, Rigaku, Japan). The Raman spectra were measured by using a LabRAM HR Evolution Raman spectrometer (HORIBA, France, 532 nm excitation) from 50 to 1200 cm^−1^ at room temperature. The morphology observation, high‐resolution lattice fringe imaging, selected area electron diffraction (SAED) and energy dispersive X‐ray spectroscopy (EDXS) were performed on a field‐emission transmission electron microscope (TEM, Talos F200X, FEI, USA) operated at 200 kV. The domain structure was observed by a high‐angle annular dark‐field scanning transmission electron microscopy (HAADF‐STEM, JEM ARM 200F, JEOL, Japan). The valence states of Ba, Ti and O were analyzed by XPS (ESCALAB 250Xi, ThermoFisher Scientific, USA). The EPR spectra were taken with a Bruker A300‐10/12 spectrometer (Germany) at a temperature of 77 K.

### Dielectric and Impedance Measurements

The temperature dependences of dielectric constant and loss were measured using an LCR meter (Agilent E4294A, Santa Clara, CA, USA) in the temperature range of −60–180 °C (at a heating rate of 3 °C min^−1^). The DBS was measured by a commercial electrometer (GIV‐010, Gogo Instruments Technology, Shanghai, China) in the DC voltage range of 0–10 kV and at the voltage elevation rate of 300 V s^−1^. Impedance spectra were measured by using an impedance analyzer (TH2838A, Tonghui Electronics Co., Ltd, China).

### Ferroelectric Measurements

P‐E loops and fatigue testing were measured at 10 Hz by using a ferroelectric testing system (WGCM20‐630F, PolyK Technologies, LLC State College, PA, USA).

### Charge‐Discharge Measurements

A commercial charge‐discharge platform (CFD‐003, Gogo Instruments Technology, Shanghai, China) with a high‐voltage vacuum relay (enduring high current up to 100 A), a current probe, a digital storage oscilloscope with 5 GSa s^−1^ sampling rate and a load resistance (200 Ω) was used to perform charge‐discharge experiments.

## Conflict of Interest

The authors declare no conflict of interest.

## Supporting information

Supporting Information

## Data Availability

The data that support the findings of this study are available from the corresponding author upon reasonable request.
