# Supplementary material for: Boosting Energy Storage Performance of Glass Ceramics via Modulating Defect Formation During Crystallization
Source: Adv Sci (Weinh). 2023 Dec 8;11(7):2307011. doi: 10.1002/advs.202307011 (PMC10953718; doi:10.1002/advs.202307011)
Supplement: Supplementary file 1 — Supporting Information [file ADVS-11-2307011-s001.pdf]

ADVANCED SCIENCE
Open Access

Supporting Information

for *Adv. Sci.*, DOI 10.1002/advs.202307011

Boosting Energy Storage Performance of Glass Ceramics via Modulating Defect Formation During Crystallization

Fei Shang, Juwen Wei, Jiwen Xu, Haibo Zhang, Yang Xia, Guisheng Zhu, Kunpeng Jiang, Guohua Chen*, Zuoguang Ye* and Huarui Xu**

Supporting Information

Boosting energy storage performance of glass ceramics via modulating defect formation during crystallization

Fei Shang, Juwen Wei, Jiwen Xu, Haibo Zhang, Yang Xia, Guisheng Zhu, Kunpeng Jiang, Guohua Chen*, Zuoguang Ye* and Huarui Xu**

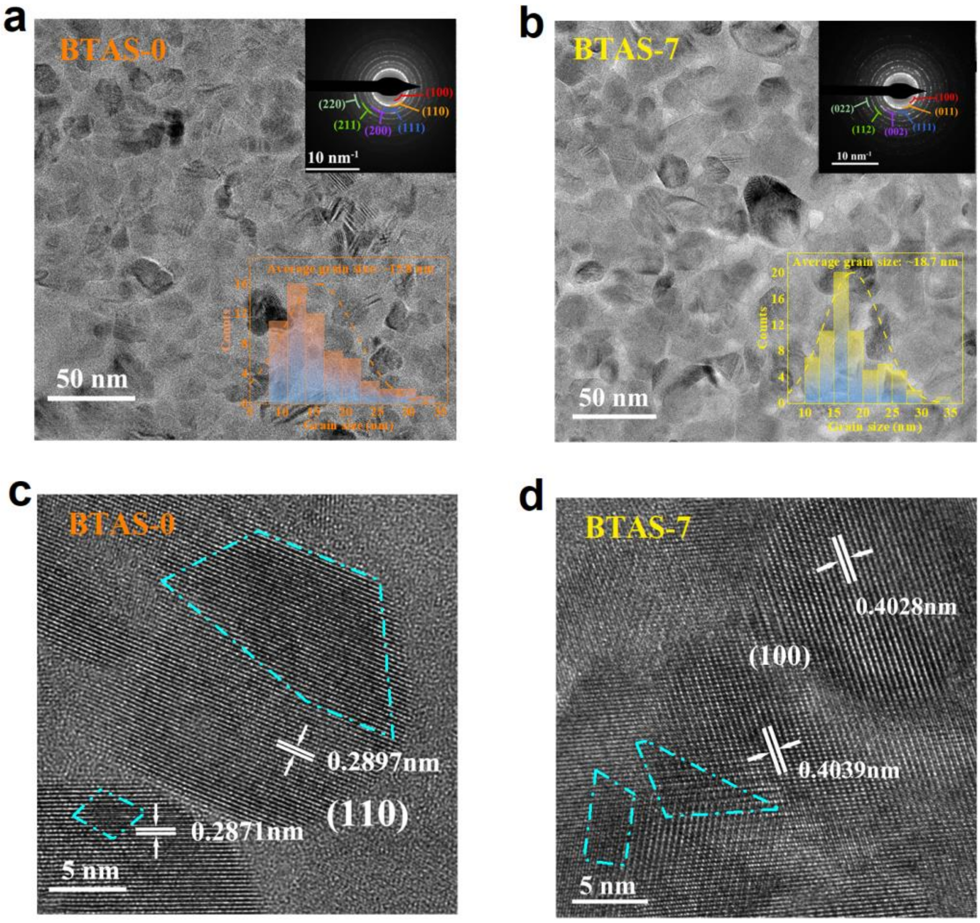

Figure S1. Morphology observed by HRTEM of **a** BTAS-0 and **b** BTAS-7 glass ceramics. The insets are corresponding SAED patterns and the grain size distribution histograms. Lattice fringes of **c** BTAS-0 and **d** BTAS-7 glass ceramics.

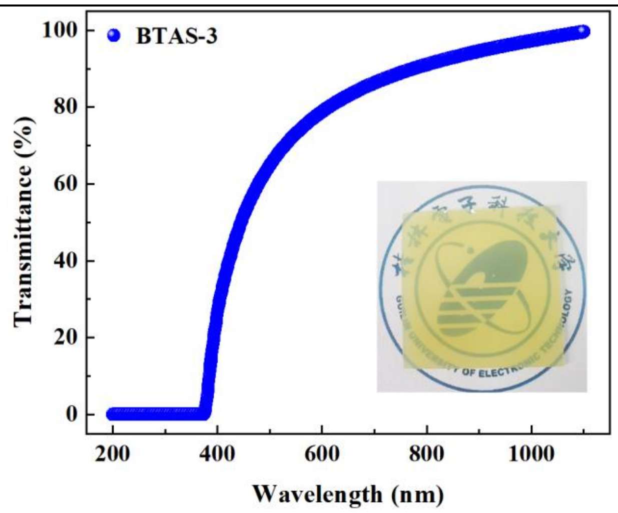

Figure S2. Optical transmittance of the BTAS-3 glass ceramic with a thickness of 0.15 mm. The inset is the photo of the BTAS-3 glass ceramic with a size of 2×2 cm.

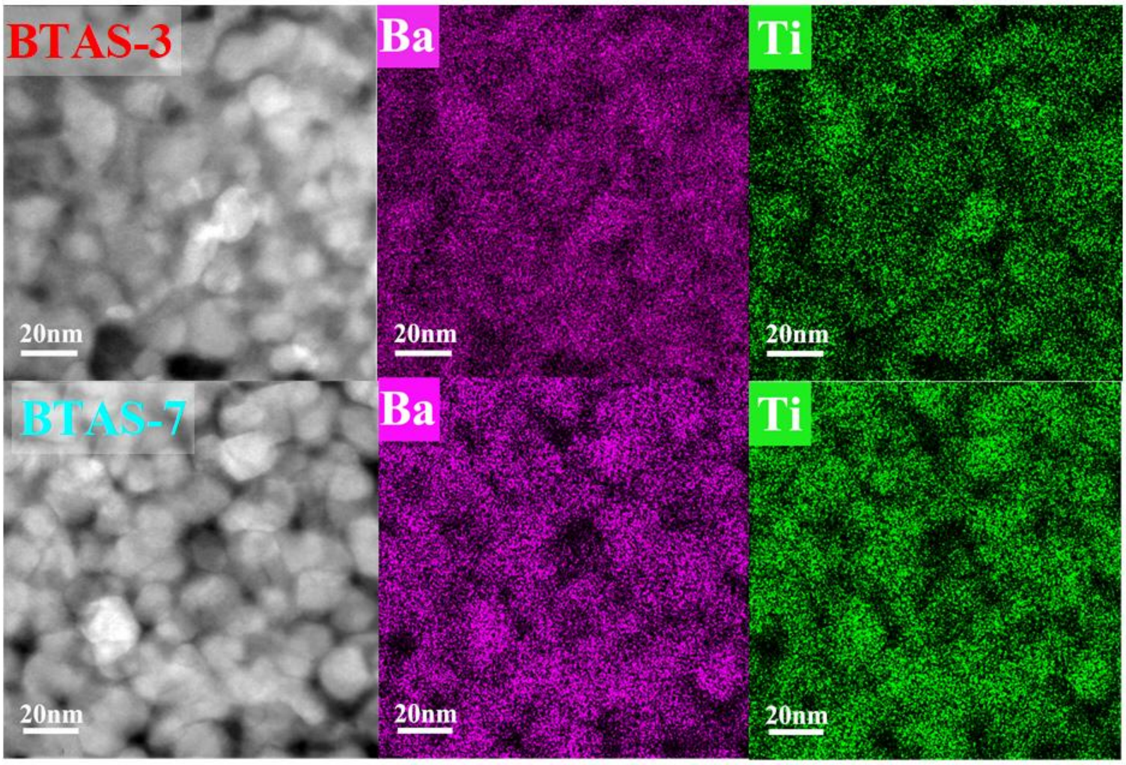

Figure S3. HAADF-HRTEM photos and EDXS element-mapping results of BTAS-3 and BTAS-7 glass ceramics.

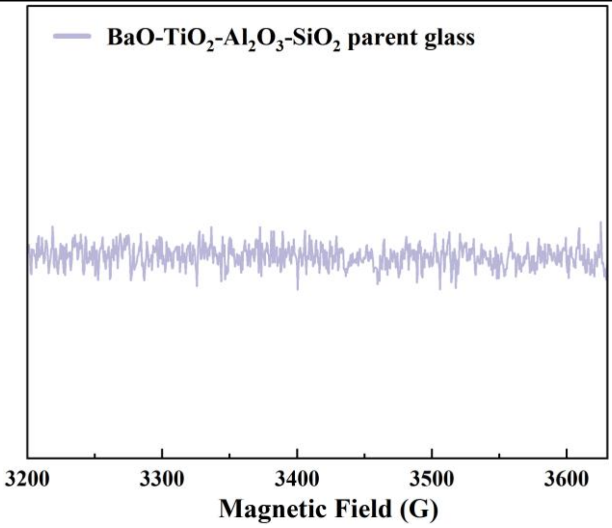

Figure S4. The first-derivative EPR spectra of BaO-TiO_2-Al_2O_3-SiO_2 parent glass.

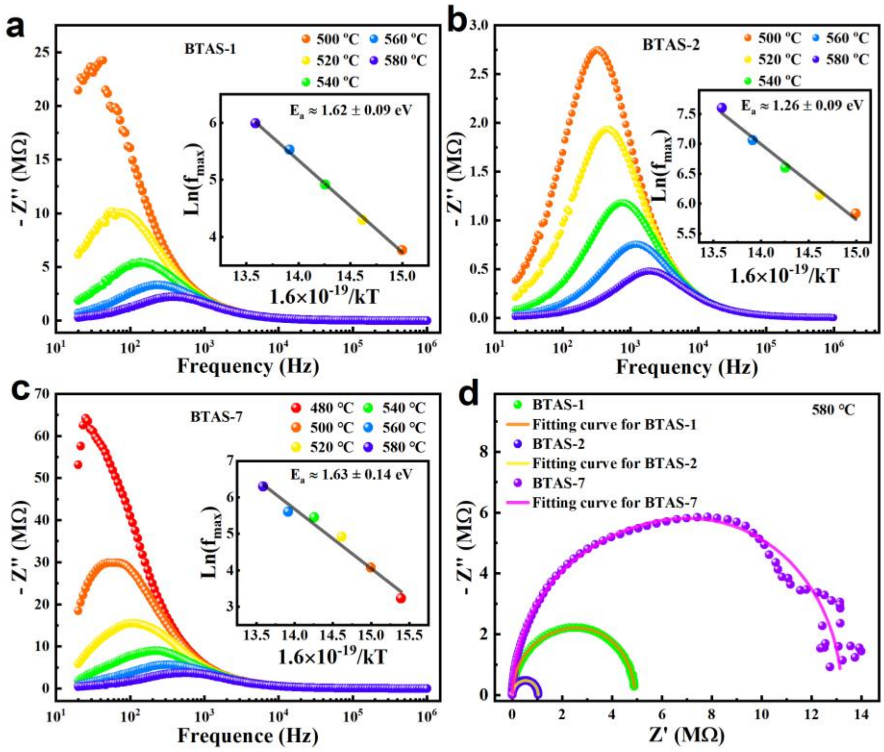

Figure S5. -Z″ versus frequency curves under different temperatures of **a** BTAS-1, **b** BTAS-2, **c** BTAS-7. **d** Cole-Cole plots of BTAS-1, BTAS-2 and BTAS-7 at 580 °C.

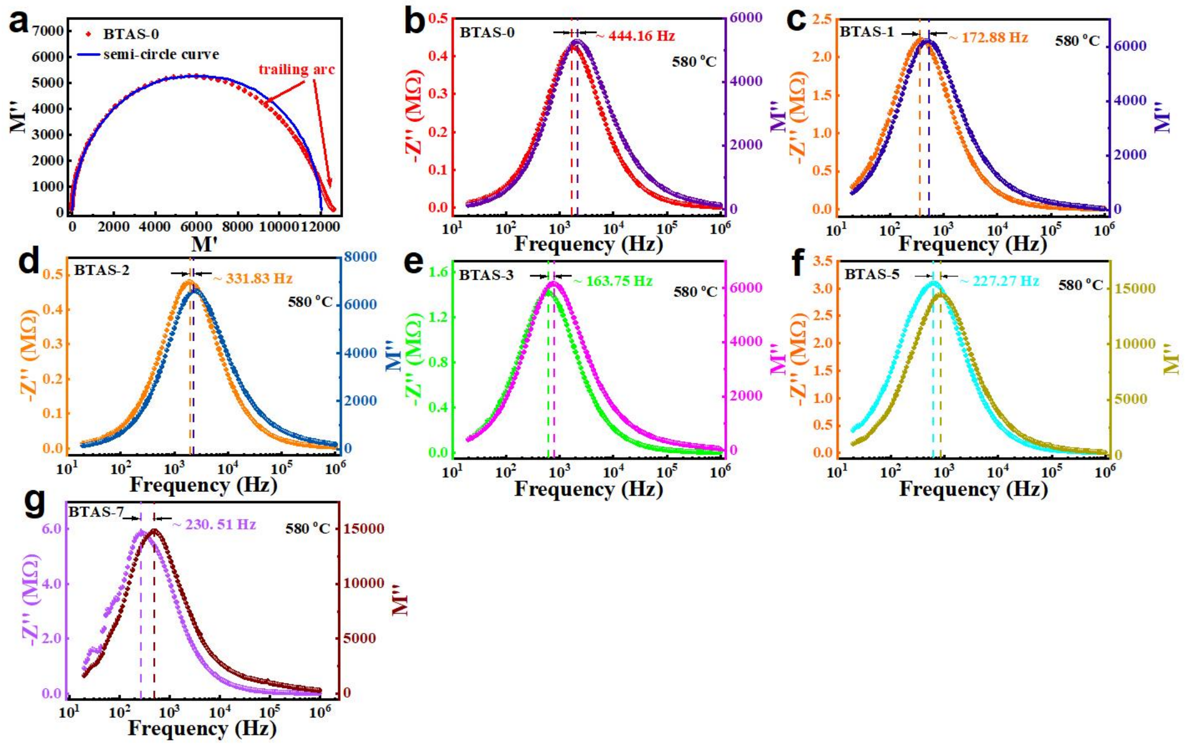

Figure S6. a Modulus spectrum of BTAS-0 glass ceramics. -Z″ and M″ versus frequency plots at 580 °C for **b** BTAS-0, **c** BTAS-1, **d** BTAS-2, **e** BTAS-3, **f** BTAS-5 and g BTAS-7 glass ceramics.

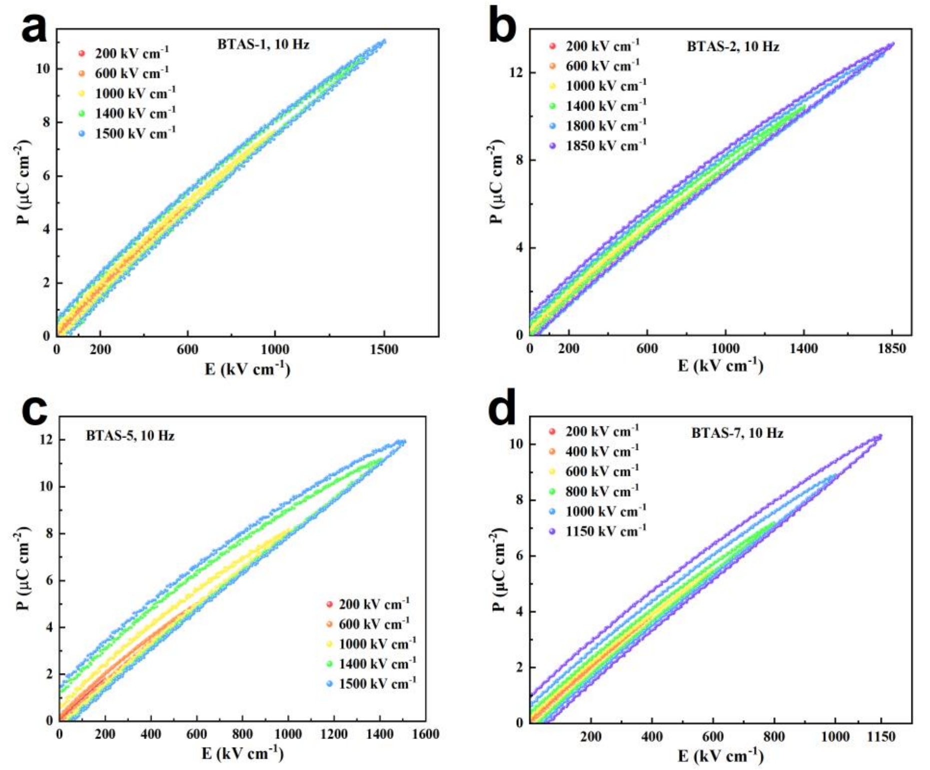

Figure S7. P-E loops at a frequency of 10 Hz of **a** BTAS-1, **b** BTAS-2, **c** BTAS-5, and **d** BTAS-7 glass ceramics with a thickness of 0.05 ± 0.01 mm and an electrode diameter of 1.5-2.0 mm.

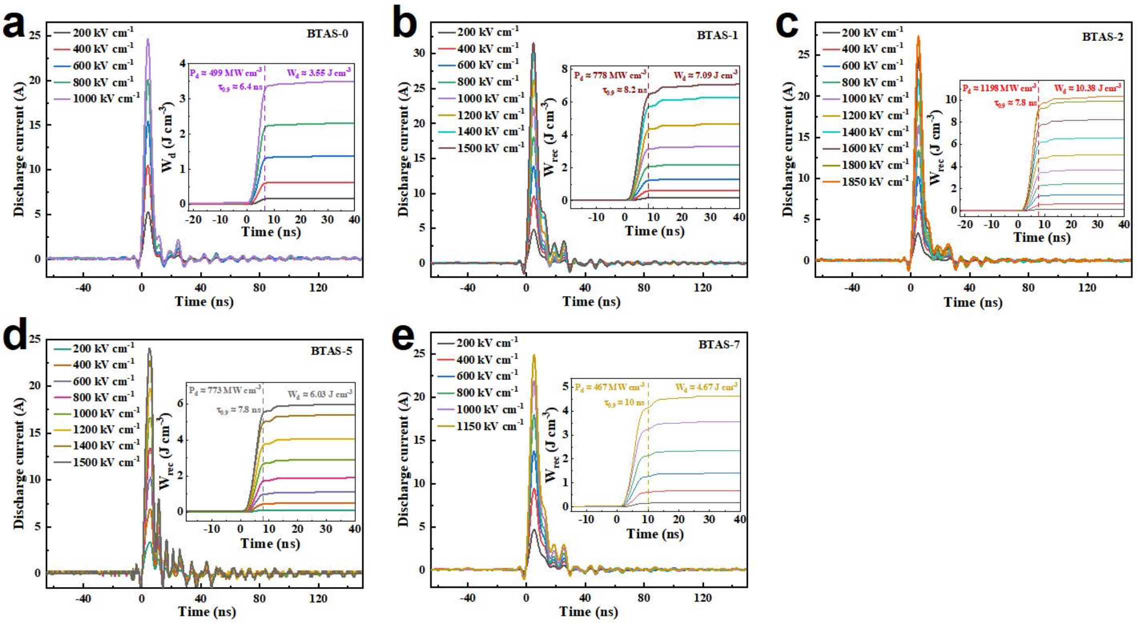

Figure S8. The time dependent curves of discharge current of **a** BTAS-0, **b** BTAS-1, **c** BTAS-2, **d** BTAS-5, and **e** BTAS-7 glass ceramics. The insets correspond to the W_d versus time curve and P_d values.

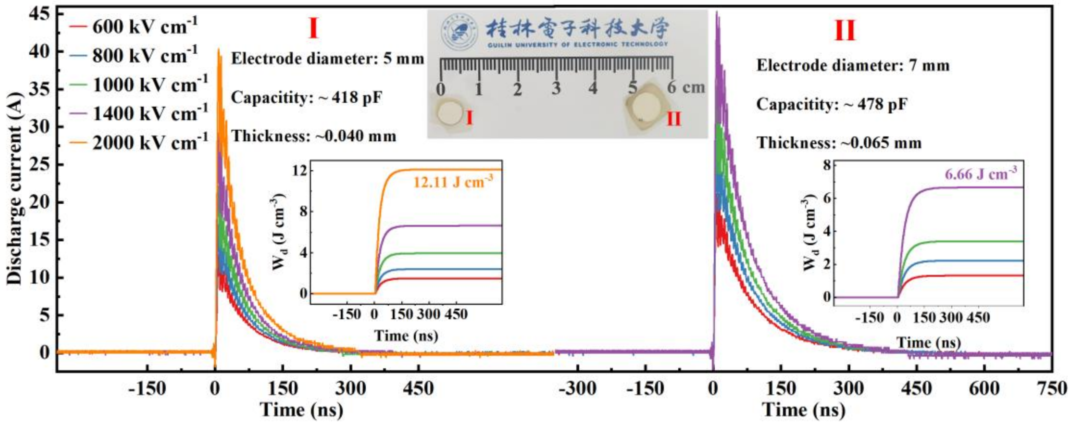

Figure S9. The curves of discharge current versus time of BTAS-3 glass ceramics with large electrode diameter of I ~ 5 mm and II ~7 mm under different charging electric field strength. The insets are the corresponding W_d values.

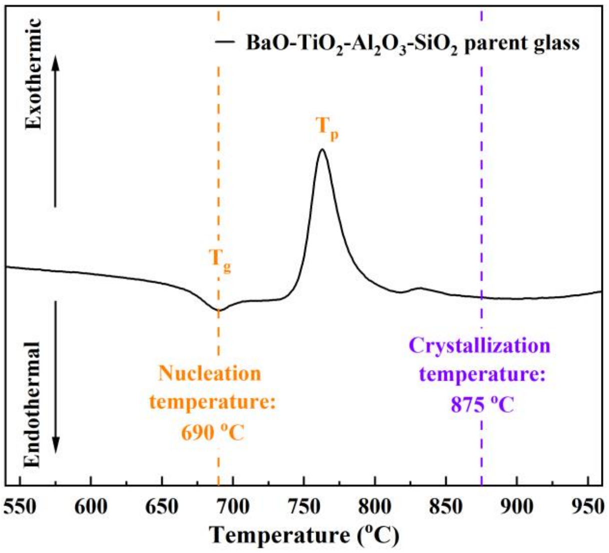

Figure S10. DSC curve of BaO-TiO_2-Al_2O_3-SiO_2 parent glass.
